# Better Mechanisms Are Needed to Oversee HREC Reviews

**DOI:** 10.1093/phe/phac010

**Published:** 2022-09-02

**Authors:** Lisa Eckstein, Rebekah McWhirter, Cameron Stewart

**Affiliations:** Faculty of Law, College of Arts, Law and Education, University of Tasmania, Australia; Bellberry Ltd, Eastwood SA, Australia; School of Medicine, Faculty of Health, Deakin University, Australia; Sydney Health Law, Sydney Law School, University of Sydney, Australia

## Abstract

Hawe *et al*. raise concerns about Human Research Ethics Committees (HRECs) taking a risk-averse and litigation-sensitive approach to ethical review of research proposals. HRECs are tasked with reviewing proposals for compliance with the *National Statement on Ethical Conduct in Human Research* for the purpose of promoting the welfare of participants. While these guidelines intentionally include a significant degree of discretion in HREC decision making, there is also evidence that HRECs sometimes request changes that go beyond the guidance provided by the *National Statement*. When HRECs request changes outside their remit, inconsistencies between individual HRECs become more common, contributing to delays in ethical review and reducing the quality of HREC decision making. Improvements to the HREC regulatory system are needed to promote transparency and accountability.

Hawe *et al.* (2022) provide a critique of the Human Research Ethics Committee (HREC) review of their citizen science pilot project. The project sought to obtain photographs and information from breastfeeding women on the facilities available for breastfeeding and expressing and storing breastmilk in Australian workplaces. The authors attributed the low recruitment rate to HREC requirements for participants to seek employer consent for any workplace photographs, or, alternatively, to agree to bear the risk of submitting a photograph without permission. The HREC also made changes to recruitment materials to formalise the language. Hawe *et al*. propose a disconnect between HRECs and social science research in particular, and suggest that the reviewing HREC erred in its risk/benefit analysis of the study.

Rather than engaging directly with questions about the studies risks and benefits, this Commentary seeks to clarify the regulatory role assigned to HRECs in Australia, and how this role can be improved through additional transparency and accountability measures.

## HREC Role Under Australian Research Regulations

Unlike some other countries, Australia does not mandate HREC review of research under legislation (with the exception of some limited areas).^1^ Instead, review requirements are specified through the *National Statement on Ethical Conduct in Human Research* (‘National Statement’), issued by the National Health and Medical Research Council (NHMRC), the Australian Research Council and Universities Australia. Compliance with the National Statement for all research involving humans is a precondition for institutions to receive research grants from major Australian funding bodies.

The National Statement includes conditions for the ethical review of research proposals. In particular, all research involving more than low risk must be reviewed by an HREC that is constituted and functioning in accordance with the National Statement (2018, ch 5.1.24). This includes minimum membership requirements, and ‘access to the expertise necessary to enable it to address the ethical issues arising from the categories of research it is likely to consider’ (ch 5.1.33). Each HREC should have ‘working procedures to promote good ethical review’, including record keeping and complaint handling processes (ch 5.1.37).

While existing data is limited, there is some evidence that Australian HRECs are highly engaged in their review functions. Brandenburg *et al.* (2021) report in their study of one Australian HREC that 83% of applications in the study period triggered requests for further information, and 17% had more than one request. While the majority of requests related to minor administrative changes, 79% related to amendments to consent forms, and over 50% were concerned with data collection; study procedures; general ethical considerations; recruitment and consent; site, setting or patient pool; research design and methodology and data management and security.

## HRECs Exercise a Significant Degree of Discretionary Power

The National Statement anticipates a degree of HREC discretion in applying its criteria. In particular, the introduction to Chapter 5.6 (Handling Complaints) states that:

There can be justifiable differences of opinion as to whether a research proposal meets the requirements of this National Statement. For this reason, while this chapter provides for complaints about the process of review, it does not provide for appeals by researchers against a final decision to reject a proposal.

This recognition of ‘justifiable differences’ reflects valid—and likely inevitable—divergence in how individuals and groups judge whether a research proposal satisfies criteria of the National Statement. For example, Hawe *et al*. dispute the HREC’s risk-benefit calculus, stating that:

the small possibility that a woman would be admonished for taking a photo of an office, lounge area, or toilet cubicle (in contravention of any workplace photography policy) has to be balanced against the more certain probability that she would feel demeaned or embarrassed to have to ask permission to do so. (Hawe *et al.* 2022, p.3)

This may be a reasonable assessment of whether the ‘potential benefits justify any risks involved in the research’ (NHMRC, 2018, ch 2.1). However, there are sound reasons why the National Statement requires a risk assessment by an independent HREC as well as by researchers. Most relevantly, risks and benefits are not objective concepts capable of precise measurement. Rather, they involve ‘normative judgments about the magnitude of respective harms and benefits, should they occur, as well as how much value the research data would have for society at large’ (Eckstein, 2015, p. 70). Moreover, people who are invested in the benefits of a research project are likely to assess its risks as being lower than others. An HREC therefore may legitimately differ from researchers (and from other HRECs) in the weight it places on the risk of submitting photographs of workplaces without consent, and on how this risk compares with research benefits.

However, not all HREC decisions are, or should be, legitimised by the National Statement. Rather, a reasonable benchmark is that an HREC must ‘objectively assess the proposal against the principles in the National Statement’ (Pieper and Thomson, 2013, p. 109). Reviewers also must ensure that they have incorporated ‘appropriate expertise related to relevant methods or areas of practice’ (NHMRC, 2018, ch 3.1). Together, these limit the scope of acceptable HREC determinations. A reasonable analogy is the Australian system for merits review of administrative decisions, and the requirements that a decision-maker act rationally within the scope of discretion, with procedural fairness, without ‘errors of fact,’ and by giving appropriate weight to the evidence (Cane, 2010). It is at least arguable that in the case reported by Hawe *et al*., the HREC has placed too much weight on the relatively low probability and magnitude of risks associated with unconsented photograph submission, and insufficient weight on the implications of requiring workplace consent on recruitment viability.

## Some HREC Requirements Fall Outside the Remit Provided by the National Statement

Some of the requirements laid out by the reviewing HREC described by Hawe *et al*. also appear to fall outside the National Statement remit; in particular, based on the formality of language in study materials.

The National Statement does not include any requirement about the way that information about participation should be expressed, or even that it be presented in writing at all. Rather, the materials should allow potential participants to attain ‘an adequate understanding of the purpose, methods, demands, risks and potential benefits of the research’(NHMRC, 2018, ch 2.2.2). The National Statement explicitly endorses presenting information ‘in ways suitable to each participant’ (ch 2.2.3, 5.2.17) and consent expressed in a way that reflects the ‘nature, complexity and level of risk of the research’ as well as ‘the participant’s personal and cultural circumstances’ (ch 2.2.5). Therefore, an HREC may request revisions if the material is expressed in a way that is likely to compromise the ability of potential participants to understand the nature, risks, and benefits of the research. But requesting revision solely on the basis of perceived informality goes beyond the National Statement, and arguably reduces compliance with the requirement to facilitate comprehension. Previously, researchers have reported feeling bound to use participant information and consent document templates or specific wording provided by HRECs in order to gain ethical approval, indicating that HREC overreach of this kind is not unusual (McWhirter and Eckstein, 2018).

Ethical review by HRECs is often criticised for being ‘rules-based’, reducing the process to a ‘tick box’ exercise (Dawson *et al.*, 2019). However, the rules being applied seem to be occasionally of HRECs’ own devising. When HREC assessments deviate from National Statement guidelines, the potential for inconsistency between HREC decisions arises. This raises particular challenges for research teams working across multiple jurisdictions, who require approval from multiple HRECs simultaneously.

## HRECs Need More Effective Oversight

Given HRECs essential role in the ethical governance of research in Australia, it is surprising that they lack basic transparency and accountability mechanisms. HRECs have some reporting obligations to the NHMRC regarding administrative matters (such as annual reporting on activity; memberships) or on substantive matters (such as when consent is waived for access to personal data). But these do not really go to ensuring the quality of decision-making.

Any regulatory mechanism should be both transparent (reasons for decisions are made clear to those affected) and accountable (the behaviour of a decision-maker is justified by reference to normative standards). The only current method of transparency is the freedom-of-information process, but requests have met with variable results when applied to HRECs (Raven v University of Sydney (2015); Whiteley and Curtin University of Technology (2008)). The only other real process for review is via a complaint back to the HREC itself or its administering institution.

More can be done to improve both transparency and accountability. For one, HRECs could provide reasons for their decisions, to be deposited in a database available to other HRECs, thereby encouraging consistency and improving the quality of decision-making. More radically, HRECs could be subject to judicial review, meaning their decisions could be reviewed by administrative decisions tribunals, broadly on the grounds that decisions were beyond power, procedurally unfair, or irrational. While this approach has not yet been taken, in principle, HRECs appear to be a form of regulatory decision-making and, as such, should be subject to these basic principles of fairness. These issues warrant consideration as a component of the development of a National Accreditation Scheme for National Mutual Acceptance Human Research Ethics Committees.^2^

## Conclusions: It Is Time to Explore Further Options for Transparency and Accountability

It is time to reconsider the HREC regulatory system to improve accountability and transparency. There are risks in adopting such mechanisms—potentially increasing delays and costs and the risk of litigious behaviour. We need to decide whether an increase in regulatory burden on HRECs could be justified by gains in the quality of decision-making. That calculus will require more research into the current behaviours of Australian HRECs and their decision-making: a notoriously difficult domain to explore.

## Footnotes

1. Areas in which HREC review is mandated include research requesting waivers of consent for the use of personal information, health research involving incapacitated persons under some state and territory guardianship laws and clinical trials involving the use of unapproved therapeutic goods.

2. Australian Commission on Safety and Quality in Health Care, ‘Consultation: Strengthening mutual acceptance of ethical review – Development of a National Accreditation Scheme for National Mutual Acceptance (NMA) Human Research Ethics Committees and Expansion of the NMA Scheme’ https://www.safetyandquality.gov.au/our-work/health-and-human-research/consultation-strengthening-mutual-acceptance-ethical-review-development-national-accreditation-scheme-national-mutual-acceptance-nma-human-research-ethics-committees-and-expansion-nma-scheme.
